# Disease Prediction Using Graph Machine Learning Based on Electronic Health Data: A Review of Approaches and Trends

**DOI:** 10.3390/healthcare11071031

**Published:** 2023-04-04

**Authors:** Haohui Lu, Shahadat Uddin

**Affiliations:** School of Project Management, Faculty of Engineering, The University of Sydney, Forest Lodge, Sydney, NSW 2037, Australia; haohui.lu@sydney.edu.au

**Keywords:** disease prediction, deep learning, electronic health data, graph machine learning, machine learning

## Abstract

Graph machine-learning (ML) methods have recently attracted great attention and have made significant progress in graph applications. To date, most graph ML approaches have been evaluated on social networks, but they have not been comprehensively reviewed in the health informatics domain. Herein, a review of graph ML methods and their applications in the disease prediction domain based on electronic health data is presented in this study from two levels: node classification and link prediction. Commonly used graph ML approaches for these two levels are shallow embedding and graph neural networks (GNN). This study performs comprehensive research to identify articles that applied or proposed graph ML models on disease prediction using electronic health data. We considered journals and conferences from four digital library databases (i.e., PubMed, Scopus, ACM digital library, and IEEEXplore). Based on the identified articles, we review the present status of and trends in graph ML approaches for disease prediction using electronic health data. Even though GNN-based models have achieved outstanding results compared with the traditional ML methods in a wide range of disease prediction tasks, they still confront interpretability and dynamic graph challenges. Though the disease prediction field using ML techniques is still emerging, GNN-based models have the potential to be an excellent approach for disease prediction, which can be used in medical diagnosis, treatment, and the prognosis of diseases.

## 1. Introduction

Electronic health data are computerised medical records for patients that contain information about healthcare entities. These data refer to a patient’s diseases or conditions and are recorded in electronic systems, with the primary goal of delivering healthcare and related services [1]. Administrative healthcare data, administrative claim data, computerised claim data, digital health records, or electronic health records are all terms that are used to describe electronic health data [2]. Electronic health data are rapidly being used for modelling and decision making in the healthcare research sector. These types of data are used for more than record-keeping in healthcare research, e.g., analysing healthcare utilisation, monitoring hospital care network effectiveness, and developing predictive models for disease prediction [2].

Machine-learning (ML) and deep-learning (DL) approaches have recently been increasingly applied in data-driven healthcare research. In terms of disease risk predictive models, many supervised ML algorithms have been used for risk assessments [3]. Likewise, DL methods have resulted in significant advances in health informatics [4]. Such models can effectively capture the intricate relationships between high-dimensional features via hierarchical levels of manipulation when used to train a predictive model [5]. For instance, the convolutional neural network (CNN) performs exceptionally well in visual medical image analyses [6]. Recurrent neural networks give exceptional accuracy in language processing through recurrent neural network architecture [4].

Nevertheless, traditional ML and DL methods explain regular Euclidean spatial data, such as medical images and medical records. The number of neighbour nodes of each node is stable in these data, indicating high translation invariance. However, there are many irregular data structures, such as patient networks [7], disease networks [8], biomedical knowledge graphs [9], chemical molecular structures [10], and gene interaction networks [11]. Graphs have irregular sizes and shapes, and they lack translation invariance. Therefore, traditional ML approaches based on normal grid-like structures cannot be used on graphs. As a result of the increasing amount of non-Euclidean data represented by graph structures, there has been an increase in interest in applying graph ML algorithms to graph-structured data. Researchers are beginning to focus on graph-structured data processing and analysis. Efforts to generalise machine-learning methods to non-Euclidean structured data have been made throughout the literature. Many methods in different graph-embedding levels have emerged, such as hand-crafted features, random walk-based techniques, and Graph Neural Networks (GNN). Hand-crafted techniques are primarily used to extract features from networks [12], which are later used to train ML classifiers for disease prediction. Random walk-based techniques, which are graph-embedding methods for mapping nodes into a low-dimensional space, are an effective solution for graph-related downstream tasks [13]. Graph neural networks are a DL method that performs inference on graph-based data [13].

### 1.1. Comparisons with the Existing Literature Reviews

Some studies have been conducted to compare supervised ML and deep-learning methods for disease prediction. Ravì et al. [4] evaluated various DL approaches for health informatics. Their research focused on critical DL applications in translational bioinformatics and medical imaging using different artificial neural networks. Min et al. [14] reviewed the performances of different state-of-the-art deep-learning methods in bioinformatics and provided future research directions. Uddin et al. [3] reviewed traditional machine-learning methods comprehensively and compared their performance in disease prediction. However, these reviews were focused on regular Euclidean spatial data. Few researchers have recently carried out GNN-based review studies using graph-structured data in bioinformatics [15,16] and medical diagnosis [17,18,19] that are based on graph-structured data. As can be seen, research on machine-learning approaches is being conducted from Euclidean spatial data to graph-structured data.

### 1.2. Motivations and Contributions

This study primarily focuses on those articles for review that used electronic health data in the disease prediction domain. It does not emphasise studies [17,18,19] that used graph-structured data from other medical sources, such as clinical data and longitudinal patient survey data. Meanwhile, Waikhom and Patgiri [20] reviewed the literature on using graph neural networks in various learning paradigms, including addressing the common formatting of graphical information and general standards or schemas that exist for the construction of graphical knowledge. However, no study in the present literature reviews disease prediction using graph ML approaches based on electronic health data. Overall, the following contributions are made by this study:

1. We review and classify different levels of graph machine-learning approaches.
2. The applications of disease prediction in different graph ML approaches are summarised.
3. We highlight the shortcomings in the present research, pointing to future research directions and opportunities.

## 2. Overview and Search Strategy

**Figure 1:**
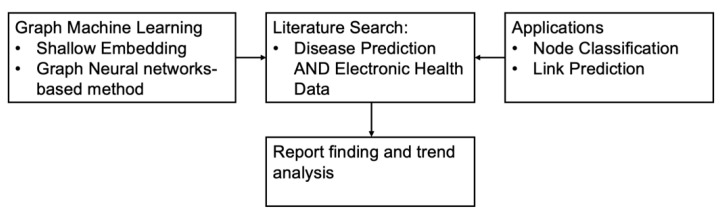
Overview of the study.

Figure 1 illustrates an overview of this study. According to this figure, a literature search is conducted based on the studies that addressed the disease prediction problem using electronic health data. Further, in doing this search, this study excludes articles that do not use any of the two graph ML algorithms (i.e., shallow embedding and graph neural network-based methods) and are neither in the application areas of node classification nor link prediction. Finally, we report the findings of this study based on the reviewed literature and trend analysis. Each of these four framework sections is further detailed in later sections of this article.

**Figure 2:**
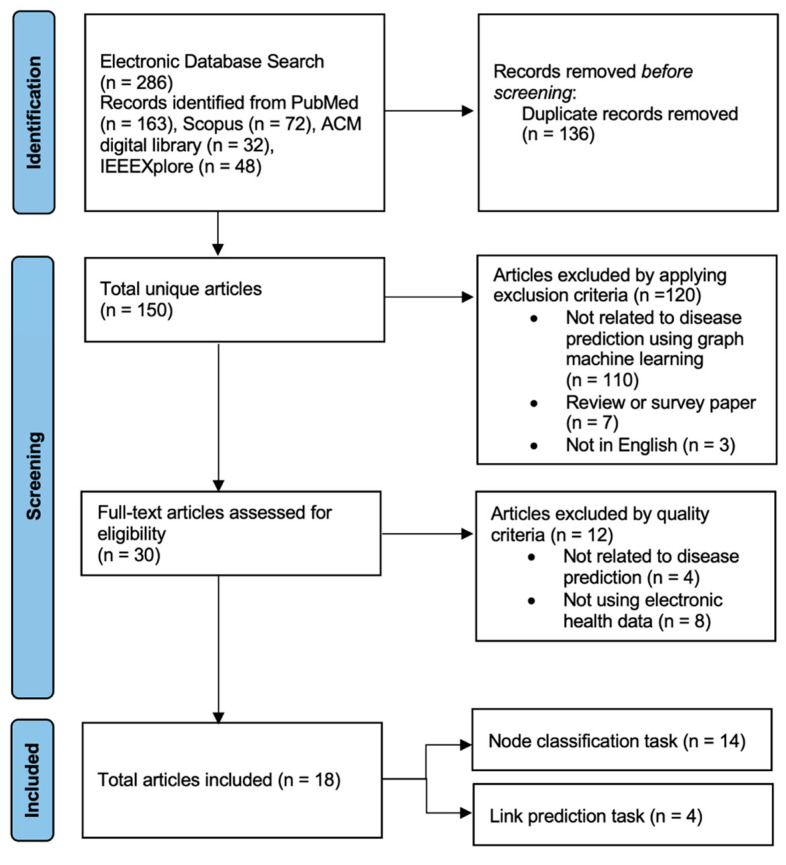
Article selection flowchart.

We searched extensively to identify articles that used the graph machine-learning method to predict diseases using electronic health data. High-quality and highly cited journals and conference proceedings were sourced from PubMed, Scopus, ACM digital library, and IEEEXplore. PubMed is a free publishing search engine that primarily includes citation data for biomedical and life science literature. It contains more than 30 million citations from MEDLINE, biomedical journals, and online books [21]. Scopus has the most peer-reviewed literature, scientific journals, books, and conference proceedings [22]. The ACM Digital Library is a searchable database of bibliographic data and full-text articles from journals and conference proceedings [23]. The IEEE Xplore database has the highest quality technical literature in engineering and technology [24]. This study’s search strategy included five keywords. They are disease prediction, graph machine learning, graph neural network, graph convolutional network, and electronic health data. We considered the full article (i.e., title, abstract, and entire body of the article) for searching. Since keywords appeared in various synonyms, quotation marks are not appended to this search query. Thus, the search string used in this study was: (disease prediction AND electronic health data) AND (graph machine learning OR graph neural network OR graph convolutional network). We further considered abbreviations and commonly used synonyms for each of these five keywords in our search using the logical OR function. For example, we considered GNN and GNNs as synonyms for the graph neural network keyword. Figure 2 illustrates the entire search approach followed in this study, how we located 18 articles to review, and the trend analysis.

## 3. Graph Machine-Learning Approaches

Graph machine learning is based on learning effective feature representations of nodes [25]. This section describes the most recent graph ML approaches, categorised into two main classes: shallow embedding methods and graph neural network-based methods. These two classes have sub-classes, as described in the following section.

### 3.1. Shallow Embedding

**Figure 3:**
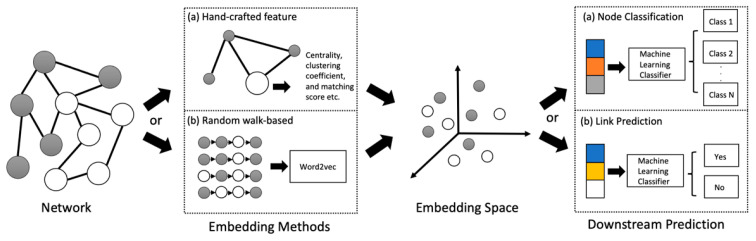
Shallow embedding methods. Graph-embedding methods extract low-dimensional node representations from the network, which are then used as features to train specific classifiers for node classification or link prediction. For (a) hand-crafted approaches, the features generated by the network are used as input to train machine-learning models. For (b) random walk-based techniques, random walks are utilised to generate node sequences. Afterwards, these sequences were fed into the word2vec [26] to extract node representations.

The semantics of domain data in a data type are captured by a shallow embedding, which offers a defined interpretation. However, shallow embedding methods can only learn and return the embedding values for their learned input data. For unseen data, obtaining the embedding vector is impossible. We categorise the shallow embedding methods into the hand-crafted feature and random walk-based methods. Figure 3 shows a high-level illustration of shallow embedding methods.

#### 3.1.1. Hand-Crafted Features

Heuristics and statistics have been developed to characterise graphs, nodes, and edges [27]. For example, various centrality measurements capture different characteristics of graphs’ connections. The betweenness centrality, for example, evaluates how many shortest paths a particular node has between pairs of other nodes [28]. The closeness centrality indicates how closely a node is linked to all other nodes [29]. Furthermore, the clustering coefficient of a node reflects how tightly its neighbours are related to form a complete graph at the node level [30]. These manually extracted graph features, commonly known as hand-crafted features, can be used to generate node representations for downstream prediction using machine-learning classifiers. There are various methods, such as kernel based (i.e., support vector machine), regression based (i.e., logistic regression), and tree based (i.e., decision tree), for such downstream predictions and classifications.

#### 3.1.2. Random Walk-Based Methods

Random walks are used to capture structural relationships between nodes in graph theory. The principle is that the distance between node representations in the embedding space should correspond to a graph distance measurement, quantified here as the frequency with which a particular node is visited in random walks originating from another node [25]. Particularly, given a graph and a beginning node, this technique randomly selects one of the node’s neighbours and moves to that neighbour. This technique will continue until node sequences are obtained. Afterwards, the word2vec, which is the method to generate word vectors by distributed numerical representations of word features [26], is used to learn embeddings from the node sequences that have been generated. This method preserves structural and topological information as latent features.

The initial work in random walk on the graph is DeepWalk [25]. It employs a randomised path-traversing method to reveal localised network topologies. It achieves this by converting random pathways into sequences, which are then used to train an unsupervised learning method for determining the most similar terms to a given word called Skip-Gram [26]. The Skip-Gram model is used to predict the next word in the sentence by maximising the co-occurrence probability of words that appear within a phrase’s frame. It can predict the next word in the phrase. Then, node2vec is employed to resolve the bias of uniformly random walks used in Deepwalk. Later, Grover and Leskovec [31] presented this node2vec model to introduce another random walk technique that balances breadth-first and depth-first searches. As a result, the sampled paths encode global and local proximities. At the same time, the embeddings generated from random walk-based methods can also be used as the input for downstream prediction.

### 3.2. Graph Neural Network-Based Methods

Gori et al. [32] introduced the concept of Graph Neural Networks (GNNs). They stated that information is naturally represented graphically. Therefore, a model can be developed to process graph structure data directly. Later, Scarselli et al. [33] expanded on this concept and demonstrated that GNNs produce considerably better outcomes than previous ML and deep-learning approaches by iteratively exploiting graph topological information. After this, there were many studies on the variants of GNN architectures, such as Graph Convolutional Networks [34], GraphSAGE [35], and Graph Isomorphism Networks [36]. GNN models have achieved excellent performance in various domains, because they extract features based on the structure of the data and allow for automated feature extraction from raw inputs rather than hand-crafted features [37]. Currently, the research direction and application domains of GNNs have considerably increased due to the growing interest in graph structure data mining in different research areas, such as predicting the properties of chemical molecules [38], applications in natural language processing [39], and predicting adverse drug reaction signals [40].

**Figure 4:**
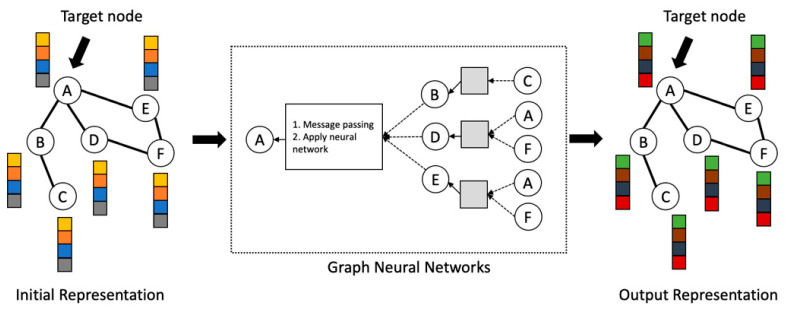
High-level illustration of graph neural network methods.

GNNs are a form of neural network that use a sequence of local message aggregation and propagation phases for graph modelling. Figure 4 denotes a high-level illustration of graph neural network methods. They can produce vector representations of graph components that capture the graph network topology and node feature [41]. The concepts of GNN are introduced as follows: Given a pair of node u and v in Graph G, the propagation at layer l is:

(1) $$h_{u}^{\left(l\right)}=UPD\left(AGG\left(MSG\left(h_{u}^{\left(l-1\right)},h_{v}^{\left(l-1\right)}\right)|\;v\in N_{u}\right),h_{u}^{\left(l-1\right)}\right)$$

where UPD denotes a non-linear function to update node embeddings, AGG is neighbourhood aggregation, and MSG is message passing. $h_{u}^{\left(l\right)}$ represents the state vector of node u at l layer, and $N_{u}$ is the immediate neighbourhood of node u.

The GNN model is a high-performing architecture for graph-structured data [33]. However, this GNN model has several limitations [37]. This model employs the same parameters in each iteration as a hierarchical feature-extraction approach. This approach is computationally expensive, since it spreads information from neighbours via a neural network until it achieves a stable fixed state to learn the node’s representation. Furthermore, several relevant features on the edges could not be successfully modelled in this approach. As a result, various variants of GNN have been developed to overcome the original GNN model shortcomings.

#### 3.2.1. Graph Convolutional Networks

Graph Convolutional Networks (GCNs) extend convolution from the Euclidean domain to the graph domain [34]. The convolution operation of GCNs is converted from Euclidean to non-Euclidean space [42]. GCNs learn a mapping function by inspecting neighbouring nodes, which can generate a new node representation by combining the information from neighbouring nodes with its feature information [33]. There are two types of existing GCN models: spectral-based [34,43,44] and spatial-based GCNs [38,45].

Spectral-based GCNs exploit the normalised Laplacian matrix of the graph and graph Fourier transform to transfer a graph’s non-Euclidean structure to a Euclidean space for convolution operations. A fixed convolutional kernel cannot be implemented on a graph, since the number of neighbours of each node is not fixed. Still, the convolutional operation can be performed when the graph-structured data are converted to the frequency domain. Given the feature vector of all nodes $x\in {\mathbb{R}}^{n}$ and a graph filter $g\in {\mathbb{R}}^{n\times d}$, the graph convolution between the two signals is:

(2) $$x*g=U\left(U^{T}x⊙U^{T}g\right)$$

where U is the eigenvector matrix. ⊙ is the Hadamard product.

If $g_{\theta }=diag\left(\theta \right),$

(3) $$x*g_{\theta }=Ug_{\theta }\left(\Lambda \right)U^{T}x$$

where Λ is a diagonal matrix of its eigenvalues, and θ is the parameter to be learned.

The above is the first generation of a GCN model based on spectral data proposed by Bruna et al. [46]. However, the computational cost is significantly high due to matrix-vector multiplication. Defferrard et al. [43] presented a model called Chebnet to overcome this limitation. Their model redefined the graph filter with Chebyshev polynomials $T_{k}\left(x\right)$ [47]. The operation is defined as:

(4) $$x*g_{\theta }\approx U(\overset{k-1}{\underset{k=0}{\sum }}\theta_{k}\;T_{k}(\tilde{L}))x$$

where $\tilde{L}$ is a diagonal matrix of scaled eigenvalues, and the Chebyshev polynomial is $T_{k}\left(x\right)=2T_{k-1}\left(x\right)-T_{k-2}\left(x\right)$ with $T_{0}\left(x\right)=1$ and $T_{1}\left(x\right)=x$.

ChebNet is not required to calculate the Laplacian matrix’s eigenvectors, which reduces the computational cost. Further, Kipf and Welling [34] truncated the Chebyshev polynomial to one time and proposed a model called GCN. This model might be useful in resolving overfitting by reducing the number of operations at each layer. The operation is as follows:

(5) $$x*g_{\theta }=\sigma \left({\tilde{D}}^{-\frac{1}{2}}\tilde{A}{\tilde{D}}^{-\frac{1}{2}}H^{\left(l\right)}W^{\left(l\right)}\right)$$

where $\tilde{A}\;$ is the adjacency matrix A for added self-connections I. $\tilde{D}\;$ is the diagonal node degree matrix of $\tilde{A}$. $H^{\left(l\right)}$ is the feature representations. $W^{\left(l\right)}$ is a learnable weight matrix, and $\sigma \left(\cdot \right)$ is an activation function.

The spatial-based GCN approach begins with the node domain and aggregates each core node and its neighbouring nodes along the edge. This operation is comparable to a CNN. These convolution methods extract the node or pixel’s neighbour information to produce the feature representation of a node in a network or a pixel in an image. However, the nodes in a graph are unordered. Gilmer et al. [48] proposed a unified framework of spatial-based GCNs, named the Message Passing Neural Network (MPNN). The MPNN is based on message aggregation between nodes and information combination. The formula is as follows:

(6) $$Aggregation:m_{u}^{k}=\underset{u\in N\left(u\right)}{\sum }M_{k}(h_{u}^{k-1},h_{v}^{k-1},e_{uv})$$

(7) $$Combination:h_{u}^{k}=U_{k}\left(h_{u}^{k-1},m_{u}^{k}\right)$$

where $e_{uv}$ is the feature representation of the edge between node u and v, $M_{k}\left(\cdot \right)$ is the aggregation function, and $U_{k}\left(\cdot \right)$ is the combination function.

However, the MPNN is computationally expensive when the number of neighbours of a node is large. Hamilton et al. [35] proposed a model called GraphSAGE. To adapt to the application on large-scale networks, it randomly samples the neighbouring nodes so that each node’s neighbouring nodes are fewer than the set number of samples. The following is the graph convolution operation:

(8) $$h_{u}^{k}=\sigma \left(W^{k}g_{k}\left(h_{u}^{k-1},h_{v}^{k-1},\forall v\in S_{N\left(u\right)}\right)\right)$$

where $g_{k}$ is the aggregation function, which can be mean, long short-term memory (LSTM), or pooling. $S_{N\left(u\right)}$ is a random sampling result of the node u′s neighbours.

#### 3.2.2. Graph Attention Networks

Many sequence-based activities make extensive use of the attention mechanism. Attention is a component of network design responsible for controlling and quantifying dependency. Veličković et al. [49] proposed the Graph Attention Network (GAT), a GNN variant that adds the attention mechanism into the propagation phases. The attention coefficient of edges u and v is represented by $\alpha_{u,v}$, and the equation is as follows:

(9) $$\alpha_{u,v}=\frac{exp\left(LeakyReLU\left(a^{T}\left[Wh_{u}∥Wh_{v}\right]\right)\right)}{\sum_{k\in N_{u}}exp(LeakyReLU(a^{T}\left[Wh_{u}∥Wh_{v}\right]))}$$

where $N_{u}$ is the neighbourhoods of node u in the graph, the input node features are denoted as $h=\left\{h_{1},\;h_{2},\ldots ,h_{N}\right\}$, a is a trainable weight vector, $a^{T}$ is the transposition of the weight vector, W is the shared linear transformation weight matrix, and ∥ is the concatenation operation. The output features of each node are:

(10) $$h_{i}^{\prime }={∥}_{k=1}^{K}\sigma \left(\underset{v\in N_{i}}{\sum }\alpha_{uv}^{k}W^{k}h_{v}\right)$$

Alternatively, a multi-head attention mechanism, consisting of K separate attention mechanisms, can be employed to enhance the expressive ability of the attention layer. The final expression is delivered as shown below:

(11) $$h_{i}^{\prime }=\sigma \left(\frac{1}{K}\underset{K=1}{\sum }\underset{v\in N_{i}}{\sum }\alpha_{uv}^{k}W^{k}h_{v}\right)$$

where $\alpha_{uv}^{k}$ is the *k*th attention mechanism.

#### 3.2.3. Graph Auto-Encoders

The wide use of auto-encoders and their variants in unsupervised learning has resulted in a rise in the number of graph generation models. Graph auto-encoders (GAEs) learn low-dimensional latent representations of nodes in the graph domain by using GNNs as encoders. Encoders in GAEs are responsible for encoding the structural information of nodes. Decoders in GAEs aim to decode the graph’s structural information from learned latent representations [50]. Kipf and Welling [51] developed a variation graph auto-encoder (VGAE) that extended the variational auto-encoder [52] into the graph domain. As with other auto-encoders, the VGAE has two components: an encoder and a decoder. The encoder employs a GCN to map each node to a low-dimensional latent representation. Afterwards, network embedding is obtained. The decoder utilises a non-linear activation to compute the pairwise distance given the network embedding. The decoder then outputs the rebuilt adjacency matrix.

## 4. Applications in Disease Prediction

**Figure 5:**
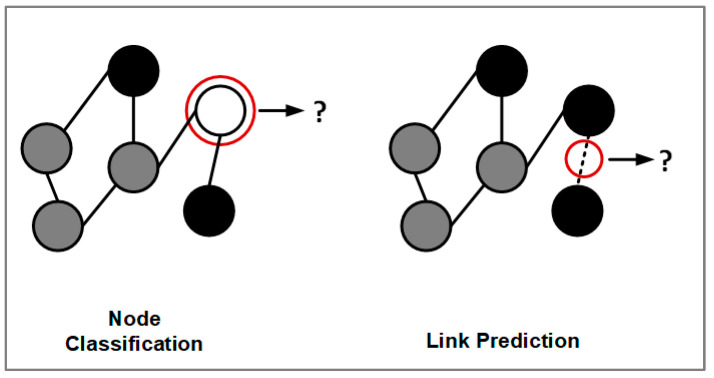
Different tasks of graph machine-learning models.

The following sections focus on the tasks of graph machine learning in the disease prediction domain. There are two levels of graph analysis tasks using electronic health data: node classification and link prediction, as illustrated in Figure 5.

### 4.1. Node Classification

Graph machine-learning methods can be used to predict an unlabelled node’s label or to classify nodes. This commonly occurs in a supervised learning environment for shallow embedding and a semi-supervised learning environment for GNN-based models. For supervised learning, shallow embedding methods are popular techniques in the disease prediction domain. These methods can learn and only return the embedding values for the learned input data. The embedding values can be used for downstream disease prediction. For example, Liu et al. [53] developed a temporal graph for patient event sequences from electronic health records. They used a network-based approach to predict the probability of heart failure onset and the risk of heart failure-related hospitalisation in individuals with chronic obstructive pulmonary disease pre-conditioning. Later, from administrative claim data, Khan et al. [8] used comorbid conditions to create a disease network for type 2 diabetic patients. They also used networks to generate features (i.e., graph node match, graph pattern match, and cluster match). Afterwards, they used these features to predict the risk of type 2 diabetes using ML classifiers. Further, Hossain et al. [54] proposed a comorbidity network to predict the risk of cardiovascular disease in type 2 diabetes patients using features generated from underlying networks. Apart from the disease network, Lu et al. [12] developed a patient network that illustrated the underlying links between health conditions for a set of patients diagnosed with the same disease. They applied ML classifiers using the network features to predict the risk of type 2 diabetes.

There are other related studies in learning graph representations in the disease prediction domain. Choi et al. [55] introduced a graph-based model that supplements electronic medical records with hierarchical information extracted from medical ontologies. Moreover, Zhang et al. [56] have introduced a Heterogeneous Convolution Neural Network (HCNN), a novel predictive learning model that depicts electronic health records as graphs with heterogeneous properties such as diagnosis, procedures, and medications. Recently, Xu et al. [57] incorporated comorbidity network embedding using a random walk-based technique on a graph that improves the performance in predicting the risk of self-harm. However, these shallow embedding methods have been widely used in predicting diseases. They can, in fact, only return a vectorial representation of the data learned during the training phase. The embedding vector for unobserved data cannot be obtained.

On the other hand, GNN-based semi-supervised learning combines the benefits of both supervised and unsupervised learning. This graph-learning approach extracts high-level node representations through information distribution, eliminating the need to label all nodes and making excellent use of certain related known information. For example, Sun et al. [58] constructed a patient record graph using medical knowledge base and electronic medical records. Then, they proposed a neural graph encoder to generate node embeddings for those graphs and predict diseases, including rare diseases for new patients. The experimental results demonstrated the state-of-the-art performance of this model in the node classification task. The node classification task using the GNN model is also popular in cancer prediction. Wang et al. [59] generated two graphs from genomic and clinical data and proposed a clinical data model based on a GCN to predict cancer survival. The GCN on the cancer sample and the sample feature matrix generation enable representation learning for all nodes in semi-supervised learning. Their work enhanced the quality of prediction when compared to previous works. Further, Gao et al. [60] presented a GNN-based framework for cancer survival prediction for the node classification task. They computed a GNN to obtain the embedding of the patient from bipartite graphs between patients and multimodal data. The output of the model is the classification of cancer patients. Another novel framework was developed by Lu and Uddin [7]. They applied the bipartite graph projection technique to generate a patient network with a weight containing latent patient relationships. Afterwards, GNN-based models are applied to predict the risk of chronic diseases. This framework can effectively learn the patterns from the network, and the performance of the GNN-based model is outstanding. Many approaches have lately used underlying spatial or temporal relationships in electronic health records to accomplish time-dependent disease prediction tasks. For example, Li et al. [61] used a GNN-based model to predict patient diagnoses by taking advantage of electronic health record data’s underlying spatial and temporal dependencies. Lastly, Zhu and Razavian [62] applied graph auto-encoders to predict Alzheimer’s disease and for other predictive tasks based on electronic health records.

### 4.2. Link Prediction

Link prediction aims to predict whether two nodes in a graph are likely to have an edge [63], which is another critical application in a graph. Predicting disease interactions from complex networks is a significant aspect of research that is becoming increasingly essential and challenging. Similarity-based methods were used to predict the risk of chronic diseases and their comorbidity. Davis et al. [64] presented the collaborative Assessment and Recommendation Engine, which is regarded as the first study to use collaborative filtering to predict disease risks. Further, Folino and Pizzuti [65] created a comorbidity network and used link prediction algorithms to infer disease connections. However, these studies focus on the similarities between diseases. Predicting comorbidity is challenging, since a multitude of circumstances can cause it. Graph ML methods have recently been applied to link prediction in disease networks. Wang et al. [66] presented a framework to predict disease risks with directed disease networks and disease risk scores. del Valle et al. [67] built a heterogeneous disease–symptom network. Afterwards, they proposed a comorbidity prediction method using Metapath2vec [68] to learn the graph embeddings. Nevertheless, these shallow embedding methods cannot generate embedding vectors for unseen data.

Recently, researchers applied GNN-based models in link prediction tasks. Wang et al. [69] used GCN on a patient–disease bipartite graph to predict the link between patients and diseases. GCN learned the target node’s representation by spreading information from neighbour nodes. The result demonstrated the proposed method had superior accuracy compared to association rules and collective matrix factorisation. Moreover, a framework combining shallow embedding and GNN-based models was proposed to predict chronic diseases and their comorbidity. The results on the administrative claim dataset reveal that it outperforms the baseline techniques, and the framework’s generalisability and performance metrics have significantly improved.

## 5. Findings

Table 1 summarises the application of different graph ML approaches on electronic health data for node classification and link prediction tasks. Applying ML approaches to electronic health data for disease risk prediction is a relatively new research direction. The first article on this subject was published in 2015. The highest number of articles (i.e., eight) was published in 2020. Researchers have adopted GNN-based methods mostly recently—all reviewed articles using GNN-based methods were published in 2020 and onwards. They reveal superior predictive performance compared with the shallow embedding approaches.

**Figure 6:**
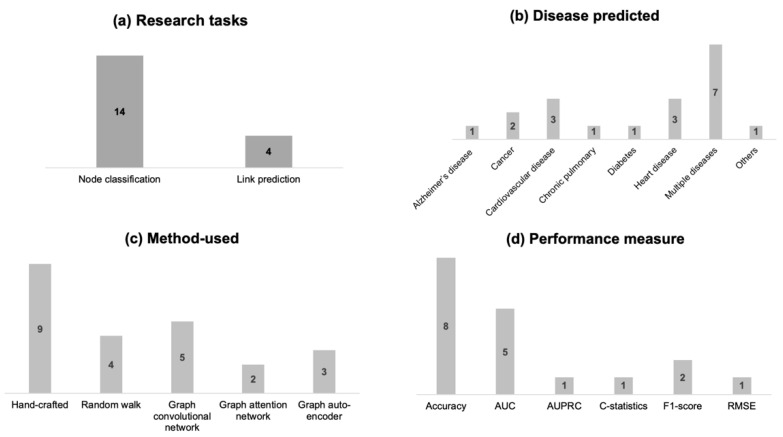
Insightful trends in applying graph machine learning for disease prediction.

Using the Table 1 data, Figure 6 presents insightful trends in applying graph machine learning for disease prediction. Researchers used graph ML approaches primarily for the node classification task (14 out of 18), as depicted in Figure 6. They tend to consider multiple diseases for risk prediction analysis (Figure 6b). Heart disease and cardiovascular disease are the two single diseases that were studied the most (three times) using graph ML algorithms and methods. Hand-crafted methods are the most used graph ML approaches (nine times) for disease prediction using electronic health data, followed by the graph convolution network (five times), as illustrated in Figure 6c. Overall, shallow embedding and GNN-based methods were used 13 and ten times, respectively. One of the reviewed articles applied both shallow embedding approaches (i.e., hand-crafted and random walk) and the GNN-based approach of GCN [7]. Few other studies used more than one shallow embedding approach and GNN-based approach [7,55,58].

**Figure 7:**
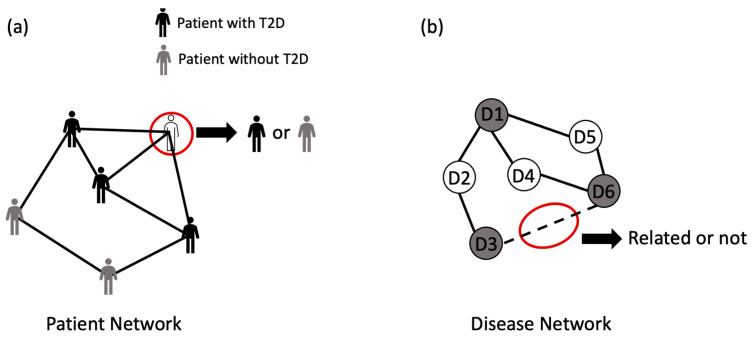
Examples of graph machine-learning tasks in two levels. (**a**) Node classification: the prediction of Type 2 diabetes (T2D) in the Patient Network. (**b**) Link Prediction: predicting the unknown link between diseases and their comorbidities through the Disease Network.

Based on the graph machine-learning methods cited in Table 1, examples of tasks in different levels are shown in Figure 7. Figure 7a shows an example of a node classification task, with the input being administrative data provided by an Australian private health fund. Following the filtering and sampling methods, two study cohorts (Type 2 diabetes (T2D) and non-T2D) were formed. Following that, a Patient Network is established. The network information and patient features are then utilised to train and test the graph machine-learning models for chronic disease prediction [12]. On the other hand, Figure 7b shows an example of link prediction. For instance, a disease network in a meta-path-based network analysis can be used to predict the probability of two diseases co-occurring [67].

**Table 1:** Summary of the reviewed articles that used graph machine learning for disease prediction using electronic health data.

Reference	Disease Predicted	Type of Data	Data Size	Task	Methods	Prediction Performance	Source Code
Liu et al. (2015) [53]	One-year hospitalisation prediction and congestive heart failure (CHF)	Real-world electronic health records over four years	319,650	Node classification	Shallow embedding (hand-crafted)	Accuracy: 76% (CHF), 65% (hospitalisation)	-
Khan et al. (2019) [8]	Type 2 diabetes	Administrative claim data from an Australian insurance company	2300	Node classification	Shallow embedding (hand-crafted)	Accuracy: 82–87% (for different machine-learning methods)	-
Hossain et al. (2020) [54]	Cardiovascular disease in patients with type 2 diabetes	Administrative claim data from an Australian insurance company	172	Node classification	Shallow embedding (hand-crafted)	Accuracy: 79–88% (for different machine-learning methods)	-
Lu et al. (2021) [12]	Type 2 diabetes	Administrative claim data from an Australian insurance company	2056	Node classification	Shallow embedding (hand-crafted)	Area under curve (AUC): 0.79–0.91 (for different machine-learning methods)	-
Choi et al. (2017) [55]	Heart failure	Three different datasets (Sutter PAMF, Medical Information Mart for Intensive Care (MIMIC)-III, and Sutter Heart failure cohort)	258,555, 7499, and 30,737, respectively	Node classification	Shallow embedding (hand-crafted and random walk)	AUC: 0.7970–0.8448 (using different training ratios)	https://github.com/mp2893/gram (accessed on 3 March 2023)
Zhang et al. (2017) [56]	Chronic disease comorbidity in patients	Anonymised electronic healthcare records data from a major medical centre	381,169	Node classification	Shallow embedding (hand-crafted)	F1 score: 0.26–0.48 (for different comorbidities)	-
Xu et al. (2020) [57]	Post-discharge self-harm incidents	Electronic healthcare records collected from Hong Kong residents	2323 self-harm samples and 46,460 counterparts	Node classification	Shallow embedding (tandom walk)	C-statistic: 0.89	-
Yang et al. (2022) [70]	Ischemic heart disease	Hospital discharge records from China	72,668	Node classification	Shallow embedding (hand-crafted)	AUC: 0.864–0.900	
Sun et al. (2020) [58]	Multiple diseases	Real-world electronic healthcare records: private patient clinical record dataset collected from local hospitals	806	Node classification	GNN based (GAT and graph auto-encoder)	F1-score: 0.457 (all diseases), 0.442 (rare diseases)	https://github.com/zhchs/Disease-Prediction-via-GCN (accessed on 3 March 2023)
Wang et al. (2020) [59]	Cancer	Electronic healthcare records collected from the US	159 for breast cancer and 160 for the lung squamous cell cancer	Node classification	GNN based (GCN)	Accuracy: 92.80% (for invasive breast carcinoma), 80.50% (lung squamous cell carcinoma)	-
Gao et al. (2020) [60]	Breast cancer	Electronic health records from Memorial Sloan Kettering Cancer Center	1903	Node classification	GNN based (graph auto-encoder)	Accuracy: 94%	-
Lu and Uddin (2021) [7]	Cardiovascular and chronic pulmonary	Administrative claim data from an Australian insurance company	2610 for the cardiovascular and 1056 for the chronic pulmonary	Node classification	GNN based (GCN and GAT)	Accuracy: 93.49% (cardiovascular disease), 89.15% (chronic pulmonary disease)	-
Li et al. (2020) [61]	Multiple diseases	A real-world longitudinal electronic health records database	7499	Node classification	GNN based (GCN)	Accuracy: 81.76%	-
Zhu and Razavian (2021) [62]	Alzheimer’s disease and multiple predictive tasks	Electronic health records, MIMIC-III, and eICU	6028, 6778, and 3250, respectively	Node classification	GNN based (graph auto-encoder)	The area under the precision-recall curve (AUPRC): 0.4580 (AD-HER), 0.7102 (MIMIC-II), and 0.3986 (eICU readmission)	https://github.com/NYUMedML/GNN_for_EHR (accessed on 3 March 2023)
Wang et al. (2020) [66]	Multiple diseases	General hospital data from two hospitals in Beijing and Shenzhen, China	7989 and 4131, respectively	Link prediction	Shallow embedding (hand-crafted)	Mean accuracy: 85.75–89.87 (for the different schemes and datasets)	-
del Valle et al. (2021) [67]	Multiple diseases	Electronic health records: DISNET	5147	Link prediction	Shallow embedding (tandom walk)	AUC: 0.74	-
Wang et al., (2020) [69]	Multiple diseases	Electronic health records from New York State Medicaid	596,574	Link prediction	GNN based (GCN)	RMSE: 0.8622	-
Lu and Uddin (2022) [71]	Multiple diseases	Administrative claim data from an Australian insurance company	19,828	Link prediction	Shallow embedding (hand-crafted and random walk) and GNN based (GCN)	AUC: 0.7964 to 0.8969.	-

## 6. Discussions and Future Directions

Disease risk predictive models assist clinicians and other stakeholders in identifying high-risk patients with few clinical resources, resulting in better individual health outcomes and lower health expenditures. The dataset, graph design, and feature selections are the most challenging aspects of establishing prediction models. Compared to electronic health data, questionnaire-based data may be less robust.

Our research dataset contained studies from 2015 to 2022, which implemented graph ML models for disease prediction using electronic health data. There has been a steady increase in the number of studies published on disease prediction using GNN-based models. The potential for using GNNs in disease prediction has been proven. GNN-based models may effectively predict outcomes when applied to unstructured grid data. GNN-based models outperform other models based on the experimental outcomes of these studies, as outlined in Table 1.

The novelty of this study can be realised from its scope, research design, and reported results. By reviewing the current literature, we first define the scope of this study. There is an absence of a comprehensive review of graph machine-learning methods for disease prediction using electronic health records. This study will fill this gap. There are review studies for disease prediction based on different machine-learning algorithms (e.g., supervised machine learning [3] and k-nearest neighbour [72]). However, there is no such study based on graph machine learning in the current literature. Second, focusing on the perspective of node classification and link prediction tasks would provide a quick update about the recent advancement in applications of these two tasks for disease risk analysis. Last but not least, the study summarises the methods used and research trends, which might be very useful to future researchers in their study design and methodology selection.

### 6.1. Benefits and Drawbacks

Table 2 outlines the strength and weaknesses of each of the graph ML approaches. Machine learning, particularly deep learning, succeeds in large-scale health informatics problems involving data in the Euclidean domain. However, extensive relationship information is retained in non-Euclidean graphs, making traditional ML approaches unsuitable for learning. Graph ML aims to embed graphs in low-dimensional spaces while retaining graph topology and node attributes. It connects graphs with contemporary ML methods and has lately attracted the interest of both the machine-learning and health informatics communities. High-quality benchmark datasets, such as ImageNet [73], are critical in machine-learning research. However, commonly used benchmarks are difficult to achieve in disease prediction using the graph machine-learning domain. For example, as indicated in Table 1, the majority of the research employed real-world electronic records. There are existing benchmark datasets (for example, MIMIC-III [74]) for disease prediction. However, they are rarely employed in the field of graph machine learning. On the other hand, most studies’ models and data are not open source. There are only three papers that provided the source code and data on GitHub, which makes reproduction difficult. Data privacy is one of the utmost concerning issues for research studies using healthcare [75]. Due to the availability of standard de-identification algorithms, health research based on electronic records is much less prone to privacy fraud.

The general disadvantage of neural networks is the black box problem. The internal operations of sophisticated algorithms are difficult to trace from the outside. It is difficult to understand how a GNN-based model reaches its conclusion. Another issue is the computational expense. Even though we are using graphs as the data structure, the computational cost will rise with each iteration and weight update during the training process. Each iteration will add more node information from the neighbourhood, increasing the number of relations and weights to calculate for each node.

### 6.2. Data Processing

The advancement of high-throughput technologies facilitates the collection of electronic health data. However, many electronic health data collections exhibit sample category imbalances. Further, the data source is electronic health data, which are sensitive to data errors in which data cleaning or imputation is also involved. Currently, the electronic health records available for research are sparse, and the format is non-standardised. Professional researchers are needed to gather more accurate data to improve the quality of electronic health data. Another limitation is the coding accuracy of electronic health data. One of the causes of variations in coding practice is the different coding policies and approaches across different legislative settings worldwide. Understandably, the corresponding health community has seriously considered this coding diversity problem over time. They are now closer to a standard coding policy than ever before.

Overall, this research provides comprehensive literature reviews of different graph ML models and their applications in the predictive disease domain using electronic health records. Because of the nature of the electronic health record, comparing the accuracy of different graph ML algorithms was only conceivable when a standard dataset was available. As a result, we concentrated solely on the literature that employed graph ML algorithms in disease prediction. According to the findings of this study, GNN-based models outperformed state-of-the-art ML algorithms. Given the GNN-based models’ exceptional ability to cope with unordered and irregular data and their simplicity and scalability, graph-based DL will play a more significant role and supplement traditional ML methods in the coming future [79].

### 6.3. Challenges and Trends

Based on current promising trends in disease prediction using graph ML approaches, we expect growth to continue, particularly for GNN-based techniques. We summarise several ongoing or prospective research directions based on the recent review results. To begin with, the majority of disease prediction methods used similarity-based approaches. The homogeneous or heterogeneous network information mostly constitutes the disease similarity, and multiple association data are extracted using graph ML models. However, no general standards or schemas exist for the creation of graphical knowledge. The development of multiple similarity networks from the data, on the other hand, would have increased the complexity of the graph ML models and led to the black box problem. Therefore, the methodologies necessary to generate an effective graph are a future research direction. More emphasis should be placed on incorporating node and edge features into the modelling processes. Further, GNN-based models can offer better interpretable analysis and visualisation, because the entities and relationships in these models frequently correlate to many types of items that exist in the real world [16]. Continued research on interpretability in graph machine models remains an important area of future research. Moreover, in addition to the diseases mentioned above, other diseases such as COVID-19 [80] and thyroid diseases [81] are currently of concern. It is also worth investigating how to use graph machine-learning techniques to predict these diseases. Lastly, as the volume of data grows, networks are not always static. Existing graph ML models were primarily concerned with static networks, whereas network evolution conditions were mostly ignored. Existing approaches must be trained again for each timestamp to learn embeddings for a dynamic network, which is computationally expensive and may not capture the temporal features. In order to cope with dynamic networks in the field of disease prediction, new graph ML approaches need to be devised.

## 7. Conclusions

An overview of various graph ML techniques in disease prediction models based on electronic health data is presented in this study. We compare different graph ML models for disease prediction at two different levels: node classification and link prediction. Specifically, we used the search strategy described in the methods section to extract the articles included. It is observed that GNN-based models have superior performance in disease prediction problems compared to traditional ML techniques. As we have shown in this study, the research in disease prediction using GNNs is growing to suggest we are on the cusp of a paradigm shift. In addition, due to their ability to cope with unordered and irregular graph data in the healthcare domain and their simplicity and scalability, GNN-based models will increasingly play a more significant role in this domain.

On the other hand, although GNN-based models have achieved outstanding performances in many disease prediction tasks, they face black box problems and dynamic graph challenges. We believe there is enormous potential to apply GNN-based models in medical diagnosis, treatment, and disease prediction. Healthcare policymakers might use the findings of this study to establish future research initiatives, and prospective future researchers might use this research to obtain an overview of the present research on disease prediction using graph ML models.

## Figures and Tables

**Table 2:** The advantage and limitations of different types of graph machine-learning models.

Graph Machine-Learning Model	Advantage	Disadvantage
Shallow embedding (hand-crafted features)	– The most basic approach, which is simple to use [13]. – Through feature engineering, this approach often allows for selecting a set of good descriptive graph properties [13].	– Computationally expensive [13]. – Not suitable for inductive applications, since shallow embedding methods are inherently transductive [50]. They cannot generate embeddings for unseen data.
Shallow embedding (deep walk based)	– When the data volume is sparse, it performs well [13]. – It can implement parallel operations and has high scalability [13].	– Unsuitable to dynamic networks [13] and inductive applications [50]. – Computationally expensive and inefficient for large graphs [13]. – Lack of shared parameters [20]. – Cannot use any node features for modelling [20].
GCNs	– Extend convolutions into graph-structured data. Unstructured grid data can be processed using GCNs [34]. – Allows for parameter sharing. – Applicable both in transductive [34] and inductive [50] settings.	– A black box technique, which is hard to interpret [76]. – Suffer from their shallow structure; for example, only two layers in Kipf and Welling’s model [34]. However, adding more graph convolution layers may hurt the performance [13].
GATs	– Can deal with input of varying sizes and can direct the model’s attention to the element most relevant to the task [13]. – More appropriate for inductive problems [49].	– Computationally expensive and more difficult to optimise [13].
Graph auto-encoder	– It can develop interpretable latent representations for undirected graphs [51]. – Learning numerous layers using a graph auto-encoder is more efficient than learning one transformation with principal component analysis [77].	– The idea of an auto-encoder cannot be straightforwardly applied, because graph-structured data are irregular [13]. – Instead of learning as much relevant information as possible, a graph auto-encoder learns to capture as much information as possible. Therefore, some useful information may be lost [78].

## Data Availability

Not applicable.

## Abbreviations

Abbreviation	Definition
AUC	Area under curve
AUPRC	The area under the precision-recall curve
CHF	Congestive heart failure
CNN	Convolutional neural network
DL	Deep learning
GAE	Graph auto-encoders
GAT	Graph attention network
GCN	Graph convolutional network
GNN	Graph neural networks
HCNN	Heterogeneous convolution neural network
MIMIC	Medical Information Mart for Intensive Care
ML	Machine learning
LSTM	Long short-term memory
T2D	Type 2 diabetes
VGAE	Variation graph auto-encoder
